# Trends in genome dynamics among major orders of insects revealed through variations in protein families

**DOI:** 10.1186/s12864-015-1771-2

**Published:** 2015-08-07

**Authors:** Nadav Rappoport, Michal Linial

**Affiliations:** School of Computer Science and Engineering, The Rachel and Selim Benin School of Computer Science and Engineering, The Hebrew University, Jerusalem, Israel; Department of Biological Chemistry, The Alexander Silberman Institute of Life Sciences, The Hebrew University of Jerusalem, Edmond J. Safra Campus, Givat Ram, Jerusalem, 91904 Israel

**Keywords:** Comparative proteomics, Genome annotation, Protein classification, Hierarchical clustering, Protein families, Arthropods, Gene novelty, Homology search, Social insects

## Abstract

**Background:**

Insects belong to a class that accounts for the majority of animals on earth. With over one million identified species, insects display a huge diversity and occupy extreme environments. At present, there are dozens of fully sequenced insect genomes that cover a range of habitats, social behavior and morphologies. In view of such diverse collection of genomes, revealing evolutionary trends and charting functional relationships of proteins remain challenging.

**Results:**

We analyzed the relatedness of 17 complete proteomes representative of proteomes from insects including louse, bee, beetle, ants, flies and mosquitoes, as well as an out-group from the crustaceans. The analyzed proteomes mostly represented the orders of Hymenoptera and Diptera. The 287,405 protein sequences from the 18 proteomes were automatically clustered into 20,933 families, including 799 singletons. A comprehensive analysis based on statistical considerations identified the families that were significantly expanded or reduced in any of the studied organisms. Among all the tested species, ants are characterized by an exceptionally high rate of family gain and loss. By assigning annotations to hundreds of species-specific families, the functional diversity among species and between the major clades (Diptera and Hymenoptera) is revealed. We found that many species-specific families are associated with receptor signaling, stress-related functions and proteases. The highest variability among insects associates with the function of transposition and nucleic acids processes (collectively coined TNAP). Specifically, the wasp and ants have an order of magnitude more TNAP families and proteins relative to species that belong to Diptera (mosquitoes and flies).

**Conclusions:**

An unsupervised clustering methodology combined with a comparative functional analysis unveiled proteomic signatures in the major clades of winged insects. We propose that the expansion of TNAP families in Hymenoptera potentially contributes to the accelerated genome dynamics that characterize the wasp and ants.

**Electronic supplementary material:**

The online version of this article (doi:10.1186/s12864-015-1771-2) contains supplementary material, which is available to authorized users.

## Background

With the maturation of sequencing technologies, we now have a large number of completely sequenced genomes. Computational and statistical tools are being developed for comparing genomes and discovering the intriguing differences in gene organization [1]. The application of such tools to Arthropod genomes has revealed genomic signatures (e.g., repeated elements, transposable elements) and conserved elements (e.g., regulatory sequences) [2–5]. A comparative genomics study of 12 Drosophilae species led to a deeper understanding of the evolutionary forces that shaped this phylogenetic branch [6].

In recent years, the number of fully sequenced genomes from insects has grown rapidly. However, genome features that contribute to the outstanding diversity among insects are only partially known [6]. *Apis mellifera*’s genome and proteome provide a glimpse of the first Hymenoptera social insect [7]. Formicidae (ants), like bees, are social animals [8] with a remarkable diversification dated from over 100 million years ago [9]. Currently, Arthropods [10, 11] are represented by tens of complete/draft genomes that cover a broad evolutionary time scale [12, 13]. Gene innovation, evolution of regulatory sequences [14], and genome dynamics were proposed in view of the ability of species to cope with extreme conditions (e.g., for the case of *Daphnia pulex* proteome [15]). Co-evolution with plants and various pathogens [16], episodes of lateral gene transfer [17] and haplodiploidy were postulated to shape the genomes of some insects [18].

It is a major computational challenge to systematically assign functional annotations to coding sequences in newly sequenced genomes [19, 20]. In this study, we investigated the benefit of combining 17 completely sequenced insect genomes as well as one crustacean (*D. pulex*) [21]). These proteomes jointly included almost 300,000 sequences. Our primary goal was to provide a comprehensive, unbiased systematic approach for partitioning insects’ proteomes to functional families. Applying routine annotation schemes (e.g., Pfam [22]) allowed assignment of molecular functions to a large fraction of the proteins. Still, no Pfam keywords were assigned for 27 % of all proteins. We took advantage of the completeness of proteomes, and quantified the variability among insects using the notion of statistically significant species-specific families. We analyzed the main evolutionary branches of insects (e.g., Diptera and Hymenoptera) in view of hundreds of cases of expansion and contraction of protein families. We postulate that variability among species with respect to their families is a good proxy for revealing the lineage-uniqueness of species.

## Results

### Clustering by protein sequence similarity distances

The protein sequences that were included in the analysis were derived from completely sequenced genomes. There are 287,405 protein sequences (72 % from UniProtKB [23] and 28 % from Hymenoptera Genome Database [24]). The two larger species groups belong to Hymenoptera (48 %) and Diptera (32 %). The number of sequences from all analyzed proteomes is summarized in Additional file 1: Table S1.

**Fig. 1:**
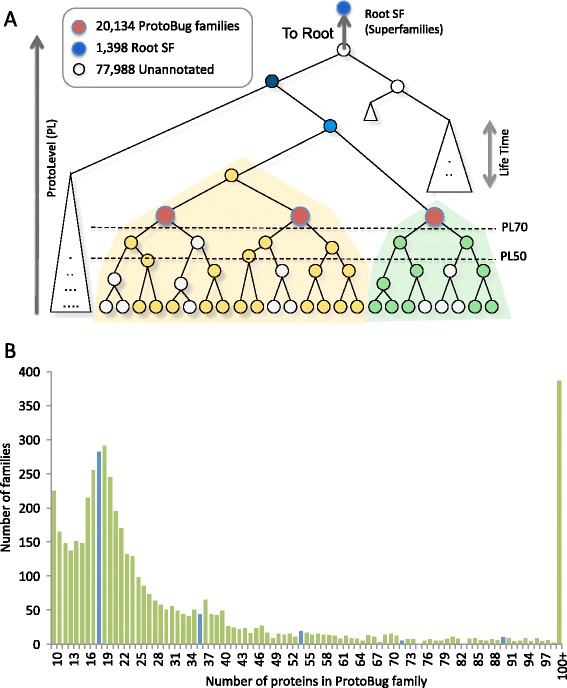
The hierarchical clustering of protein sequences from complete sequenced genomes. **a** ProtoLevel (PL) is a normalized measure for the time of clustering procedure, where all leaves and the root cluster have PL = 0 and PL = 100, respectively. Cuts at predetermined PL thresholds are shown (dashed lines). At a certain cut, the clusters are a collection of disjoint families. Higher value for PL is associated with a smaller number of protein families. Empty circles mark proteins that are unannotated by an external expert system (e.g., Pfam) but belong to the family. Root superfamilies (Root SFs) are clusters at the top of the hierarchy based on a pruning of the binary tree at PL99. The total number of ProtoBug families, Root SFs and proteins that have no external annotations is shown. **b** Size distribution of the protein families from 18 Arthropods-complete proteomes. The histogram of protein families is ranked by their sizes. The blue bars show families of size 18 and multiplications (i.e., 36, 54). All families with >100 proteins each are combined

We performed an all-against-all BLAST search for the entire set of sequences that resulted in a large distance matrix (with 8.2*E^10 E-score values). This matrix is used as input to a hierarchical clustering that is based on the ProtoNet algorithm and on a predetermined partition of the output tree [19, 25]. Fig. 1a shows the clustering scheme for all 18 analyzed species (see Methods). Notably, the representation of species among families having 18 proteins is significantly higher in view of the random expectation (*P*-value = 0.00059). Note that there are hundreds of families (coined ProtoBug families) that are very large and include at least 100 proteins each (Fig. 1b).

**Fig. 2:**
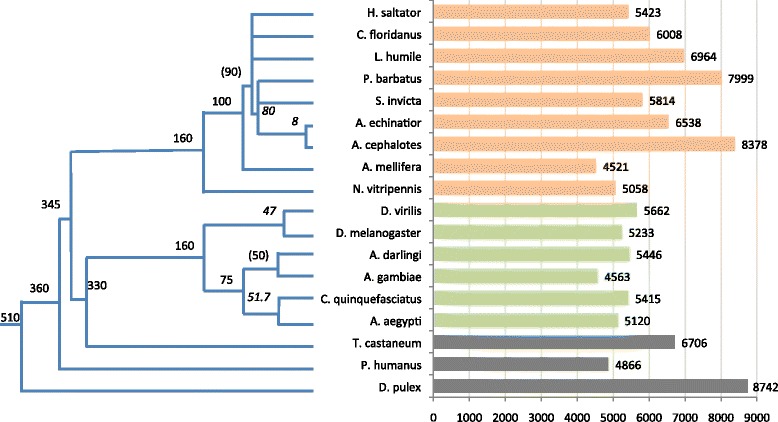
Protein families of the Arthropods complete proteomes by species-specific assignment. Each of the 18 analyzed organisms is associated with the listed number of protein families (as in Fig. 1a) along the accepted phylogenetic tree. *D. pulex* serves as an outgroup. Numbers on the nodes of the phylogenetic tree are the estimated branch length of speciation (in million years). Branch lengths are extracted from [40], TimeTree [57] (italic) and inference (numbers in parenthesis). The sources for all 18 complete proteomes and the number of families according to PL70 partition are summarized in an Additional file 1: Table S1

The clustering protocol led to 20,134 clusters (of size >1, Fig. 1a) and additional 799 singletons. These are disjoint protein families. Figure 2 shows the number of families with respect to the accepted taxonomy tree. The proteomes are partitioned on average to ~5200 families in the case of Diptera and to ~6300 families for Hymenoptera.

### Quality of annotation assignment

To assess the quality of the automatically defined protein families we assigned keywords to each protein for domains, families and repeats according to its predicted Pfam keywords. The number of proteins that remained unannotated was 77,988 (27 % of all sequences, Fig. 1a). Altogether 4,400 Pfam keywords were assigned to the 18 analyzed proteomes, and the functional coherence of each family was quantified with respect to the Pfam keywords.

Table 1 lists the largest families (>1000 proteins each) according to their size and family specificity score (see Methods). We assessed annotation quality and coherence for each of the resulting families. We found very high average specificity (0.89), confirming the quality of the unsupervised classification protocol with respect to external knowledge. As mentioned, the clustering protocol relies entirely on sequences and used no annotations or pre-knowledge. Within a family, unannotated proteins are assumed to share the same function as the annotated proteins in the family (for an inference threshold, see Methods). We refer to such inference as “annotation gain” (Table 1). Among the 20,134 disjoint protein families (>1 protein each), 4503 families have a minimal size of ≥10 proteins each. Families with a small number of proteins (<10 members) are more sensitive to noise. Therefore, the rest of the analysis focuses on families with at least 10 proteins. A comprehensive list of 3437 mapped Pfam keywords (associated with 4503 ProtoBug families, ≥10 proteins) is available in Additional file 2: Table S2.

**Table 1:** Largest families, associated Pfam keywords and family specificity

# proteins	# insects	Pfam ID	Pfam name	# TP^a^	# FP^b^	# gain^c^	Spec.
4952	18	PF13465	Zinc-finger double domain	3150	1343	459	0.701
3857	18	PF00069	Protein kinase domain	2954	809	94	0.785
2912	18	PF00089	Trypsin	2895	0	17	1.000
2646	18	PF00400	WD domain, G-beta repeat	2415	37	194	0.985
2240	18	PF07679	Immunoglobulin I-set domain	1391	632	217	0.688
1940	18	PF13855	Leucine rich repeat	1467	323	150	0.820
1860	18	PF12796	Ankyrin repeats (3 copies)	1580	165	115	0.905
1749	18	PF00067	Cytochrome P450	1694	0	55	1.000
1721	18	PF00076	RNA recognition motif. (RRM, RBD, RNP)	1520	125	76	0.924
1667	18	PF00379	Insect cuticle protein	1599	1	67	0.999
1647	18	PF00046	Homeobox domain	1400	147	100	0.905
1630	17	PF02949	7tm Odorant receptor	1507	0	123	1.000
1559	18	PF00071	Ras family	1082	445	32	0.709
1529	18	PF00001	7 tm receptor (rhodopsin family)	1417	43	69	0.971
1160	18	PF00651	BTB/POZ domain	1076	46	38	0.959
1100	18	PF00083	Sugar (and other) transporter	973	105	22	0.903

^a^ *TP* True positives, ^b^ *FP* False positives, ^c^ *gain* unannotated proteins, *Spec*. specificity

### Diversification in protein families

**Fig. 3:**
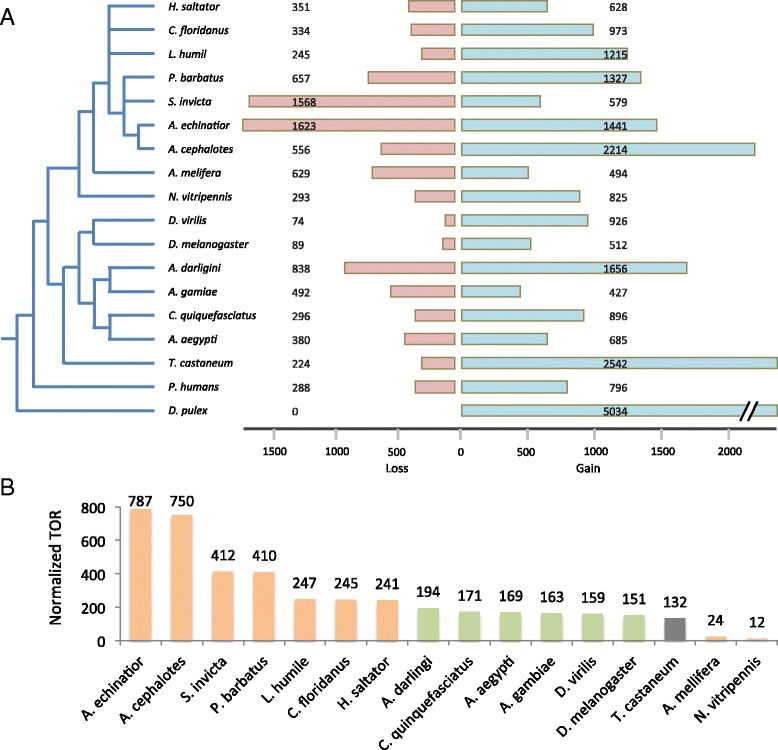
Gains and losses of protein families. **a** For each species the right bar represents the number of gained protein families, and the left bar (colored light pink), the number of losses. The highest number is associated with *D. pulex*, an outgroup species for the 17 insect proteomes. **b** Turnover rate (TOR) for all species. TOR is calculated as the sum of gains and losses from the leaf up to the root. For TOR estimation we used branch length as shown in Fig. 2. The Hymenoptera are associated with a higher overall TOR with respect to Diptera

By comparing families, we derived an indirect assessment for the divergence rate. We searched for all family-species pairs and focus on protein families where a species (or group of species) is present or absent with respect to neighboring species in the phylogenetic tree. These are assigned as family gain and family loss (see Methods). The highest number of families gained is associated with *D. pulex* (4969 families, Fig. 3a). Extreme diversification with over 2000 families gained is associated with *T. castaneum* and *A. cephalotes*. Table 2 is a sample of families that are defined as Gain and Loss (G and L, respectively) with respect to *S. invicta*. Note that many of these clusters are annotated with broad terms such as “Signal peptide” or “Transmembrane”. A minimal family size of 50 was required for determining a family loss. The list of gains and losses for all 18 species is available in Additional file 3: Table S3.

**Table 2:** Samples of gain and loss families with respect to *S. invicta*

Cluster ID	G/L^a^	Size^b^	Representative	# proteins *S. invicta*	Major annotation keyword
550959	G	19	E9J1M0_SOLIN	1	Signal Peptide
543127	G	14	E9J5N7_SOLIN	6	Signal Peptide
539306	G	69	E9J871_SOLIN	1	Poxvirus a32 protein
551666	G	10	E9INN7_SOLIN	1	Transmembrane domain
550404	G	12	E9IWW7_SOLIN	1	Toxin-like protein/Signal
543940	L	69	E2BBV3_HARSA	0	Glycosyl hydrolase family 1
552258	L	90	E2C9S9_HARSA	0	Transmembrane domain
544213	L	69	Q9VWC5_DROME	0	Skp1 family, tetramerisation
536474	L	87	Q9VA69_DROME	0	Prolyl 4-Hydroxylase alpha
552903	L	57	E2AJS9_CAMFO	0	Transglutaminase-like
544830	L	77	Q4V5X1_DROME	0	Ninjurin
553509	L	174	E2BZE5_HARSA	0	Toxin-like protein /Signal
547355	L	58	Q8MRA9_DROME	0	DDE superfamily endonuclease

^a^G/L, Gain or Loss of a family, respectively. ^b^Size, the family sizes for G and L are ≥10 and ≥50, respectively

We further estimated the dynamics at internal branch in the phylogenic tree by estimating the turnover rate (TOR, Fig. 3b). We found that TOR for the Hymenoptera clade is significantly higher with respect to Diptera (KS test’s *P*-value 0.01, Fig. 3b). Among the Hymenoptera clade, the proteomes of the different ants have in general the highest TOR. Using BadiRate tool [26] for assessing TOR, we confirmed that the higher TOR is significantly associated with the Hymenoptera with respect to the Diptera (for 11 out of 12 binary trees, see Methods).

Most families contain representatives from multiple species (i.e., homologous proteins). The number of proteins in a family indicates evolutionary events at the genome level such as gene duplication or retrovirus integration. We therefore systematically identified such events by monitoring families’ expansion and contraction for any of the analyzed species. The number of proteins in a family for a species was often skewed with respect to the number of sequences. For example, in a family annotated “7tm odorant receptor” (Table 1), there are 204, 69 and 8 proteins, from *Atta cephalotes*, *Acromyrmex echinatior* and *Pediculus humanus*, respectively. Divergence is estimated by a significant deviation in the representation of some species (calculated by the hypergeometric survival function, with a threshold of *P*-value <0.05 and corrected for multiple hypotheses, see Methods).

**Fig. 4:**
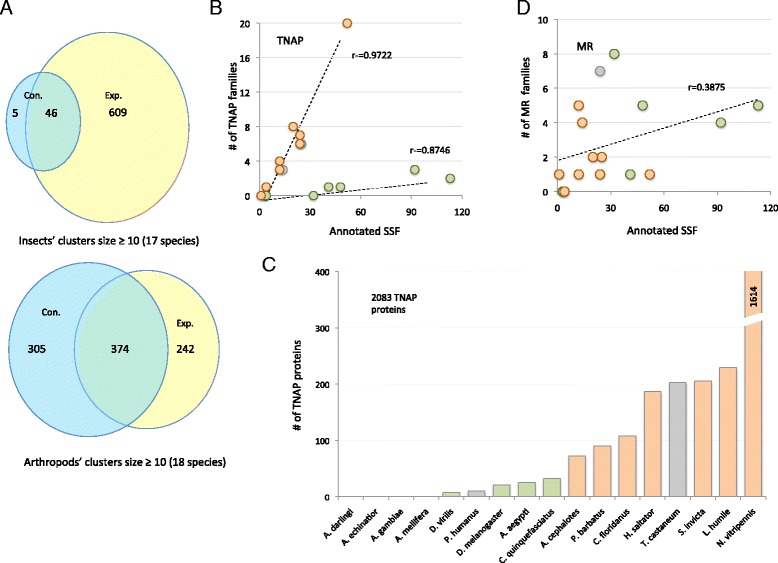
Divergence with respect to protein families. **a** Venn diagrams of protein families (≥10 proteins) with a significant expansion or contraction (right and left circles, respectively). Top: the analysis based on 17 insect proteomes. A total of 655 SSF (species-specific families) were significantly expanded in at least one species. 51 SSF were significantly contracted and 46 families intersected (i.e., at least one species was expanded and at least one contracted). Bottom: the analysis based on 18 Arthropod proteomes, 17 insects and a proteome of *D. pulex*. **b** Ratios of SSF that belong to unified set of Pfam annotations (i.e., high-level functionality) relative to all annotated SSF. The analysis for TNAP is illustrated for each of the species. Correlation lines and calculated coefficients are shown. **c** Histogram of the number of proteins that belong to TNAP for each species. **d** Identical analysis as in (**b**) for the unified functionality of membrane receptors (MR)

We collected 665 families of size ≥10 that showed an expansion for one (or more) insect species, and 51 families that have a significant contraction in at least one of the insects. Note a substantial overlap of the two lists (Fig. 4a). As could be anticipated, once the *D. pulex* is included, the number of families with significant expansion or contraction is far higher. *D. pulex* contributed an additional 339 and 102 families, for expansion and contraction, respectively (Fig. 4a, bottom).

We defined the statistically significant families of size ≥10 as SSF (species-specific families). Table 3 shows a sample of most significant (in terms of *P*-values) SSF for two insects. The SSF list for all 18 species is found in Additional file 4: Table S4.

**Table 3:** Pfam keywords for *N. vitripennis* and *S. invicta* expanded and contracted protein families

Family Size	# Prot.	Pfam ID	Pfam Name	*P*-value	E/C^a^	Species
1860	227	PF12796	Ankyrin repeats (3 copies)	1.00E-307	E	N. vitripennis
63	22	**PF00078**	Reverse transcriptase (RNA-dependent DNA polymerase)	1.00E-307	E	N. vitripennis
14	14	PF00076	RNA recognition motif. (RRM, RBD, or RNP domain)	1.00E-307	E	N. vitripennis
33	16	**PF10551**	MULE transposase domain	1.00E-307	E	N. vitripennis
58	48	PF12259	Protein of unknown function (DUF3609)	1.00E-307	E	N. vitripennis
50	29	**PF00665**	Integrase core domain	1.00E-307	E	N. vitripennis
42	24	**PF12596**	87 kDa Transposase	1.00E-307	E	S. invicta
57	25	PF05699	hAT family dimerisation domain	1.00E-307	E	S. invicta
14	11	PF00698	Acyl transferase domain	1.00E-307	E	S. invicta
464	65	**PF13359**	DDE superfamily endonuclease	1.00E-307	E	S. invicta
85	27	PF00348	Polyprenyl synthetase	1.00E-307	E	S. invicta
67	26	**PF00078**	Reverse transcriptase (RNA-dependent DNA polymerase)	1.00E-307	E	S. invicta
69	32	**PF03184**	DDE superfamily endonuclease	1.00E-307	E	S. invicta
865	0	N/A		3.28E-26	C	N. vitripennis
795	1	N/A		2.17E-22	C	N. vitripennis
4952	196	N/A		1.03E-15	C	N. vitripennis
1048	7	N/A		2.60E-15	C	S. invicta
2912	62	PF00089	Trypsin	2.72E-15	C	S. invicta
399	0	N/A		1.80E-12	C	N. vitripennis
1667	29	PF00379	Insect cuticle protein	3.97E-12	C	S. invicta
243	51	PF02949	7tm Odorant receptor	2.21E-11	E	S. invicta
67	28	**PF00078**	Reverse transcriptase (RNA-dependent DNA polymerase)	2.47E-11	E	N. vitripennis
80	29	PF05585	Putative peptidase (DUF1758)	4.04E-11	E	N. vitripennis
865	8	N/A		4.35E-11	C	S. invicta
295	150	**PF05380**	Pao retrotransposon peptidase	9.09E-11	E	N. vitripennis

Only families with *P*-value < E-10 are listed. Bold, annotations related to TNAP. ^a^E/C refers to an expanded or a contracted family

### Functional enrichment of most diverse protein families

SSF from Diptera dominated the annotated list (63 %). We limited the functional analysis to families assigned annotations (i.e., annotated SSF, see Methods). The annotated SSF accounts for 58 % of all SSF and covers 294 Pfam keywords. We observed a drastic variation in the number of annotated SSF associated with the different species. Yet, many annotations are shared by several species. For example, a family annotated “Trypsin” (2912 proteins) shows a *P*-value of 3.8E-10 and 7.2E-11 for family expansion in *Aedes aegypti* and *Anopheles gambiae*, respectively.

Inspecting the function associated with SSF allows, in an initial approximation, to postulate on the functional uniqueness of a species. Toward this goal, we mapped SSF to only a few high-level functional descriptors. We unified Pfam keywords by the Pfam Clan assignments [27]. For example, a high-level function of signaling receptors (collectively called membrane receptors, MR) includes ion channels and sensory receptors annotated as 7tm chemosensory receptor, 7tm odorant receptor, transporters and ion channels (GO:0006811, ion transport and GO:0051716 cellular response to stimulus, see Methods). Another large group of Pfam keywords is the transposition and nucleic acid processes (collectively called TNAP). The Pfam keywords that are combined by TNAP include transposase, non-specific endonucleases, viral and transposons, integrase and multiple families of reverse transcriptase (total of 23 Pfam keywords). Additionally, we confirmed the association of TNAP with the biological processes of transposition and DNA-mediated process (GO:0006313) [28].

Figure 4b shows the prevalence of TNAP families among the annotated SSF, for any of the 17 insect species. There are two distinct strong correlation lines for the annotated SSF that belong to TNAP: (i) species that belong to Hymenoptera (light orange, r = 0.972); (ii) species that belong to Diptera (light green, r = 0.875). Notice that *T. castaneum* and *P. humanus* that do not belong to either of these clades (named others, colored gray) follow the correlation line of the Hymenoptera. The fraction of TNAP families among the annotated SSF of the species that belong to Diptera accounts for only 3–4 %, while among Hymenoptera it reaches 30-40 % (Fig. 4b, a steeper correlation line). Reinforcing the partition of insects into two distinct sets is based on counting the proteins within the TNAP families (Fig. 4c). From all annotated SSF associated with TNAP, 89.3 % are from Hymenoptera and 7.6 % and 3 % are from others and Diptera, respectively. Among all insects *Nasonia vitripennis* displays an extreme TNAP expansion with 1614 proteins (Fig. 4c).

We repeated the analysis for additional high-level functionality. In the case of membrane receptor (MR) function, the two clades are inseparable (Fig. 4d). We conclude that the clear difference in the appearance of TNAP families (Fig. 4b) is not evident for MR function (Fig. 4d). Additional high-level functions such as Proteolysis (GO:0006508) and Cell-matrix adhesion (GO:0007160) failed to separate the SSFs between the two major clades of insects.

### An unbiased function view leads to biological interpretation of superfamilies

By analyzing SSF we seek functions that are associated with one (or more) species. In order to generalize the observation and to allow analysis for families that fall short when using the annotation inference protocol (see Methods, unannotated SSF), we tested all families at the highest level of the hierarchical tree. Root superfamilies (Root SFs) are the end product of the clustering protocol for merging nested families and subfamilies (Fig. 1a). The ~300,000 proteins from all 18 species are merged into 1398 Root SF clusters (excluding singletons, see Methods, Fig. 1a).

**Fig. 5:**
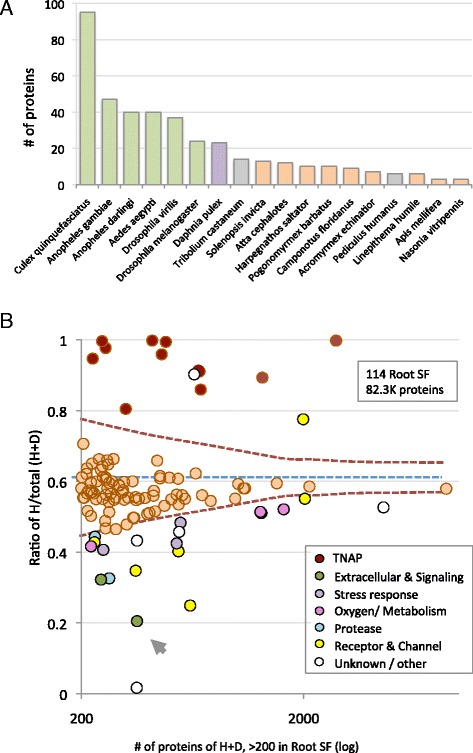
Analysis of Root superfamilies (SF). **a** Number of proteins for 18 species for a Root SF with 399 proteins annotated “Fibrinogen-beta and gamma chains, C-terminal globular domain”. The maximal number of proteins is associated with Diptera and specifically with the 4 mosquitoes. **b** 114 Root SFs that have a size of >200 proteins from Hymenoptera (H) and Diptera (D). Considering only protein from Diptera and Hymenoptera, the baseline probability for Hymenoptera proteins is 0.61 (dashed line, see Methods). A confidence threshold based on binomial distribution at *P*-value <10e-5 is shown as dashed bent lines. The high-level functionalities for expanded and contracted Root SF are color-coded. TNAP, transposition and nucleic acids processes; H, Hymenoptera; D, Diptera. The Root SF annotated Fibrinogen that is analyzed in (**a**) is marked by an arrowhead

Figure 5a shows the protein partition among the 18 species for a Root SF annotated “Fibrinogen- beta and gamma chains, C-terminal globular domain” (399 proteins). This Root SF is of very high quality (99 % selectivity, 95 % specificity and includes 87 unannotated proteins). We noted a 4:1 ratio in favor of the proteins belonging to Diptera as compared to Hymenoptera (*P*-value <1.0E-56, Fig. 5a).

Altogether there are 114 Root SFs with a minimal size of >200 proteins. A table with a summary of these 114 Root SFs is in Additional file 5: Table S5. We inspected all 114 Root SFs and exposed all Root SFs that were characterized by a skewed appearance of Hymenoptera versus Diptera proteins. When combining proteins from Hymenoptera and Diptera, the partition of the proteins is 61 % and 39 % respectively. We used this ratio as a baseline for assessing the statistical deviation. Figure 5b displays the high-level functions of Root SF that are significantly skewed for the Hymenoptera versus the Diptera (shown as symbols below and above the statistical lines). The dominant high-level functionalities include membrane receptors (MR), signaling domains, metabolic enzymes and extracellular components. The abundance of Root SFs that cover the TNAP function is outstanding in Hymenoptera (dark red symbols, Fig. 5b). Almost all statistically significant Root SFs that are dominated by Hymenoptera include TNAP functions (e.g., transposons, DDE superfamily endonuclease, DNA polymerase of organelle and viruses, helicase, exonuclease, viral function, phage integrase, Poxvirus proteins). On the other hand, Root SFs in which the proteins of Diptera versus Hymenoptera favor Diptera lack a functional coherence. Additionally, some extreme statistics are associated with extracellular localization (Fig. 5a, arrow) and membrane receptors (MR, yellow symbols).

### Traces of viral protein integration in wasp genome

**Fig. 6:**
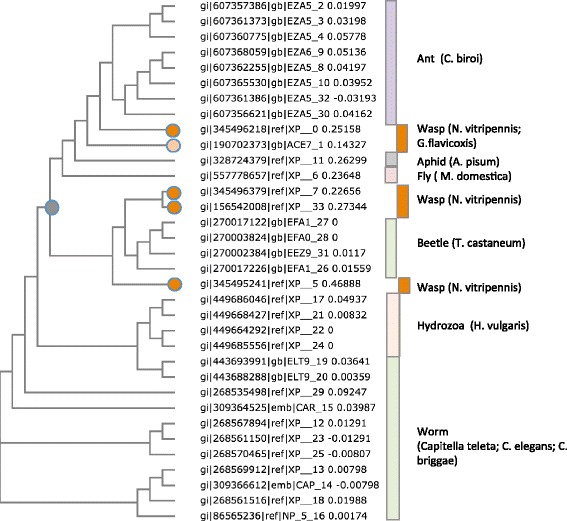
The homologues of Poxvirus A32 protein. *N. vitripennis* hypothetical protein LOC100680040 (XP_003427466.1) was used as a query to create a multiple sequence alignment. About half of the resulting proteins belong to additional insects (*C. biori*, *M. domestica*, *A. pisum* and *T. castaneum*). Appearance of *N. vitripennis* proteins in multiple nodes of the dendrogram is consistent with evolutionary episodes of spreading of the virus sequences through the parasitoid Nasonia species [64]

The potential signature for the expansion of TNAP in *Nasonia vitripennis* (Fig. 4c) was tested. One of the SSF families and a Root SF with an extremely statistically significant variability in Hymenoptera vs. Diptera is annotated “Poxvirus A32 protein”. The Poxvirus A32 protein [29] encodes a conserved ATPase that involved DNA packaging in virions of double strand (ds) viruses. Surprisingly, we identified 69 appearances of Poxvirus A32 protein with 68 of them belonging to the *N. vitripennis* (Jewel wasp). Fig. 6 shows a dendrogram for Poxvirus A32 homologues from the *N. vitripennis (*based on a query of LOC100680040, XP_003427466.1, hypothetical protein). The dendrogram shows branching to worms and hydra in addition to various insects. Proteins from *Cerapachys biroi* (clonal raider ant) and *Tribolium castaneum* are confined to a single branch of the dendrogram, while the distribution of the proteins from the parasitoid wasp is indicative of gene duplications and a high divergence rate.

## Discussion

In this study, we applied an unsupervised, sequenced-based clustering algorithm [25] for classifying the complete proteomes from Arthropods into families. The byproduct of such classification allows: (i) large-scale functional inference; (ii) quantification of the dynamics of insect proteomes; and (iii) search for evolutionary and functional insights. We will briefly discuss each of these outcomes.

### Functional inference

In this study we focused on 18 proteomes from sequenced genomes. The quality of genome assembly may lead to varying completeness of the input. However, some statistical features of the analyzed genomes are at a comparable level (e.g., protein length, number of homologues, fraction of membranous proteins and coverage by Pfam keywords). Importantly, many protein families exhibit consistent functional annotations (i.e., high specificity) (Table 1).

In this study we focused on two resolution levels of the clustering hierarchy: ~20,000 high quality families and ~1400 Root SFs (Fig. 1a). For example, the Root SF “7tm odorant receptor” shows extremely high specificity (0.996) for its annotated proteins (1906 proteins). Nevertheless, only 84 % of the included proteins have Pfam annotations. We argue that the high specificity of this Root SF with the “7tm odorant receptor” keyword allows safe inference of this keyword (total of 2258 proteins). This protocol of limited inference was successfully applied to newly sequenced genomes (see [30, 31]).

### Dynamic of proteomes

A comparative analysis for assessing the dynamics of proteomes requires knowledge of evolutionary relationships between the taxa. Establishment of social life in insects was associated with an accelerated protein evolutionary rate for only a few families [32]. For example, the Yellow gene family underwent multiple gene duplications followed by positive selection [33]. Our analysis corroborates this finding by showing a skewed appearance of the Yellow gene family proteins along the insects’ lineage. The unbiased approach presented in this study provides insights on hundreds of families.

The 18 representative species are spread along a wide range of evolutionary distance. The evolutionary history of insects’ speciation is not fully resolved [34]. The *D. melanogaster* and *D. virilis* split ~50 million years ago [35]; they were selected for our analysis to represent remote speciation in view of the 12 available Drosophilae proteomes [6]. On the other hand, the *Anopheles gambiae* and *Drosophila melanogaster* diverged about 250 million years ago. An example of recent speciation is the speciation of *Atta cephalotes* and *Acromyrmex echinatior*. These ants were estimated to have split only 8–12 million years ago [36]. Our results argue that even among such closely related species, the number of families (Fig. 2) and the number of SSF do not follow the speciation scheme (discussed in [37]). It was estimated that ∼ 4000 novel genes evolved within ants’ lineage, probably to comply with unique life styles [14]. Indeed, we observed the largest variations in families among the ant proteomes (Figs. 3, 4 and 5).

We used TOR to estimate the rate of changes in the different proteomes along the evolutionary path (see Methods). There are various known methods that use a binary ultrametric phylogenetic tree for estimating TOR [38]. As some of the branches in the phylogenetic tree (Fig. 2) are undefined, methods such as CAFE [39] and BadiRate [26] could not be directly applied (see Methods). Artificially transforming the phylogenetic tree to a binary one and applying BadiRate tool to all possible resulted trees confirmed the results presented in Fig. 3b.

We provide evidence for the different evolutionary forces for Diptera and Hymenoptera based solely on their analyzed proteomes: (i) Based on TOR (Fig. 3b), the dynamic nature of Hymenoptera proteomes is revealed. TOR is calculated for families’ gains and losses in view of the most accurate phylogenetic tree and speciation branch length [40]. (ii) Despite the excess in the number of annotated SSF from Diptera’s (63 %), only 3 % of the proteins belong to TNAP, as compared to over 30 % in the case of Hymenoptera (Fig. 4) (iii). Root SFs (having >200 proteins) cover a large fraction of all analyzed proteins (30 %). Nevertheless, almost all Root SFs (13/15) that show a significant expansion for Hymenoptera proteins (Fig. 5b, *P*-value <10e-5) share TNAP functions.

A genome-based analysis for tracing the origin and quantity of orphan genes in insects was performed [41]. This study shows that an exceptionally fast dynamic is associated with the wasp and a number of ant genomes. Creating new orphan genes is attributed to the presence of transposable elements and to the accelerated genome dynamics in the Hymenoptera clade [41]. Our unbiased approach corroborates the study of orphan genes (Figs. 5 and 6).

### Expansion in function

Within the comparative genomics paradigm, genomes carry valuable information on the ability of a species to occupy a specific ecological niche [42]. The drastic expansion of TNAP function in Hymenoptera (and others) is consistent with the accelerated genome dynamics demonstrated for ants, wasp and beetle. Gene novelty is often associated with an adaptive evolution, as illustrated for a number of proteins from the innate system [43]. The evolution of protein families acting in defense against pathogens was discussed in view of insects’ social life [44, 45]. TNAP proteins are likely to act in rearrangement, homologous and non-homologous DNA editing, integration and removal of invaders (e.g., viruses, fungi, other pathogens).

The TNAP families rely on the mapping to Pfam Clan and Gene Ontology annotation. However, the strong signal for family expansion in Hymenoptera relative to Diptera was instrumental in revealing related functions (i.e., not belonging to any predefined Pfam Clan). THAP domain (PF05485), DUF1759 and GIY-YIG catalytic domain (PF01541) are examples of such instances. Mining the literature reveals their relevance to TNAP. The THAP domain (PF05485) is shared between cellular proteins and transposases from mobile genomic parasites [46]. Similarly, the DUF1759 is related to LTR-polyproteins, or retrotransposons. The GIY-YIG domain characterizes homing endonuclease and selfish mobile elements. These enzymes catalyze the hydrolysis of genomic DNA within the cells that synthesize them. As such, homing endonucleases are implicated in driving genomes’ dynamics [47].

TNAP enables crosstalk of hosts and their pathogens (e.g., between bacteria, plant, fungi, transposable elements, viruses etc.). In accordance with our observation, transposition, viruses, and nucleic acid manipulation are critical components for the evolvement of parasitic life style in Hymenoptera [16]. The dynamic exchange of genetic material from pathogenic resource (e.g., viruses) is traceable (Fig. 6). However, the expansion in functional groups is not limited to TNAP families. The evolution of olfactory receptor family along with insect speciation has been previously reported [48]. Sensory receptors of the 7-transmembrane families underwent a large expansion in only certain insects. Such expansion has been attributed to the pressure of adapting to different environments and to a genomic drift [49].

### Arthropods’ genome diversity

The platform presented in this study is applicable to completely sequenced proteomes that cover a wide range of evolutionary time. Routinely, evolutionary trends are extracted from genomic signatures (regulatory regions, non-coding RNA, Ka/Ks ratio). We show that an approach that relies on statistical criteria for complete proteomes is valuable in detecting trends for certain branches of the Arthropod phylogenetic tree [50]. In this study, we focused only on complete proteomes. The Taxonomy Database [51] contains 2.1 million sequences from insects. Almost all sequences (99.93 %) belong to Pterygota (winged insect) and 90 % of all these sequences originated from Endopterygota (4 orders - Amphiesmenoptera, Coleoptera, Diptera and Hymenoptera). Our analysis benefits from the availability of data. Currently, several genomes from Amphiesmenoptera (moth and butterfly) are not yet included.

Similar to our findings, active DNA exchange and integration of pathogens and transposition is evident to occur in Hymenoptera at a higher rate than in other arthropods [52]. Analyzing representative proteomes from Ditrysia (silkworm *Bombyx mori* and butterfly *Danaus plexippus* [53]) show that they all share the property of TNAP expansion. In contrast, the proteome of pea aphid (*Acyrthosiphon pisum*) resembles the Diptera proteome in view of minimal abundance of proteins that belong to TNAP.

In addition to the evolutionary perspective for studying insects, insect genome dynamics impact on human health and agriculture. Insects play an essential role in pollination, but also in crop loss. Other proteomes are studied with respect to human health (e.g., malaria, trypanosomiasis). We expect the upcoming large-scale initiative of sequencing of Arthropods [54] to benefit from the presented clustering platform. The ProtoBug database as well as navigation tools are accessible in www.protobug.cs.huji.ac.il [55].

## Conclusions

We show that from an input of 300,000 insect proteins ~20,000 coherent functional families are produced by an automatic, unsupervised clustering protocol. We illustrate the strength of the statistically unsupervised approach for unveiling expansion and reduction in families with respect to specific species. We suggest that the skewed representation in species-specific families serves as a guideline for phenotypic diversity. The strongest deviation from the expected number of proteins among hundreds of families was associated with TNAP proteins that were highly enriched among Hymenoptera representatives. We suggest that this signature leads to genome dynamics and may contribute to diversity in protein functions.

## Methods

### Data preparation

We downloaded the entire proteome of *Tribolium castaneum*, *Aedes aegypti*, *Anopheles gambiae*, *Culex quinquefasciatus*, *Drosophila melanogaster*, *Drosophila virilis*, *Solenopsis invicta*, *Anopheles darlingi*, *Acromyrmex echinatior*, *Camponotus floridanus*, *Pediculus humanus*, *Harpegnathos saltator* and *Daphnia pulex* from UniProtKB [56]. Other insects’ proteomes that were not available on UniprotKB were downloaded from the Hymenoptera Genome Database [24]: *Nasonia vitripennis* (v1.2), *Linepithema humile* (v1.2), *Atta cephalotes* (v1.2), *Pogonomyrmex barbatus* (v1.2) and *Apis mellifera* (BeeBase, release 4.5). Additional file 1: (Table S1) summarizes properties of the analyzed proteomes.

The *Daphnia pulex* [21] was added as an outgroup species. We selected two drosophilae (*Drosophila melanogaster* and *Drosophila virilis*) to reduce sampling bias. The total number of proteins from the combined resources is 287,405 (206,615 from UniProtKB and 80,790 from HDB). There are 138,762 proteins that belong to Hymenoptera and 91,241 to Diptera.

The phylogenetic tree was downloaded from NCBI Taxonomy [51]. Branch lengths were extracted from TimeTree [57]. Estimated values were applied to a few undefined nodes. Most branch lengths were extracted from the revised phylogenetic tree of insects [40].

### Protein families’ clustering and annotation

Protein families were generated using hierarchical clustering [58]. We performed a BLAST search of all-against-all protein sequences, with the next non-default parameters: E-value threshold was set to 100, and maximal number of hits was limited to 1000 [59]. We noted that about a third of the proteins across all species reached that limit (1000 hits). The resulting BLAST E-values were used as distances between sequences for a bottom-up hierarchical clustering [60]. Protein families were defined as the disjoint nodes when cutting at PL70. PL70 is a threshold for cutting the hierarchical clustering where 70 % of merges are already completed [25]. At this level many merges have already occurred (measured by size), clusters are non-trivial (measured by the average E-values for all possible protein pairs in the cluster), and are stable (measured by Life Time). Life Time is the difference between ProtoLevel at creation and termination [61]). We selected PL70 (Life Time = 1.0) for the collection of families. Using more advanced thresholds (e.g., PL80) had minimal impact on the observed trends. A threshold of PL99 (Life Time = 0.5) was applied to define the Root SFs. There are 1398 such Root SFs.

We associated proteins with Pfam annotations by using Pfam Scan [22]. For each pair of protein family and annotation keywords (e.g., Cytochrome P450) we computed TP (true positive), the number of proteins that belong to the family and share this annotation; FP (false positive), the number of proteins in the family that are associated with a different annotation; FN (false negative), the number of proteins having the annotation but are not part of the subjected family. Unannotated proteins that belong to the subjected family are not listed as FP. With unannotated proteins that belong to families with at least 10 proteins, if there is a keyword with specificity > 0.2, we consider the unannotated proteins as “annotation gain.”

Unification of high-level functional annotations is based on manual inspection and Pfam Clan assignment. Transposition and nucleic acids process (TNAP) combines the following keywords: transposase, non-specific endonucleases, integrase and different families of reverse transcriptases, The Clan RNase_H (CL0219), DNA-mend (CL0382) and DNase_I-like (CL0530), enzymes that act on nucleic acids include reverse transcriptase and tranposase. Viral-related functions include His-Me_finger (CL0263), GAG-polyprotein (CL0523) and Retroviral_zf (CL0511). High-level functionality for Membrane Receptors (MR) combines Pfam GPCR_A (CL0192), Chemosens_recp (CL0176), PBP_GOBP (PF01395), transporters and Ion_channel (CL0030). Gene Onthology terms are used for high-level functionality. GOA mapping of InterPro2GO provides an elaborate mapping of GO molecular function terms and parent terms [28].

### Protein families’ gains and losses

The number of protein families for each species (a leaf in the phylogenetic tree) is calculated by the number of clusters in the hierarchical tree with at least one protein representative of that species. For internal nodes (species’ ancestors), the set of families is considered as a union of two groups: (i) families that appear in more than one immediate descendant, and (ii) families that appear in one immediate descendant and in at least one sibling of that node. After assigning the set of protein families to each node, we computed the gain and loss.

Gain of protein family is defined as the set difference between families of the node and the union of families that belong to the node’s siblings and uncles. Gain of a given node *n* (i.e., leaf for species, or an internal node in the phylogenetic tree) is defined by the following formula:

$$ gain(n)=pf(n)\backslash \left[{\displaystyle \underset{s\in siblings(n)}{\cup }pf(s)}\kern0.5em {\displaystyle \underset{u\in siblings\left( father(n)\right)}{\cup }pf(u)}\right] $$

Where *pf(n)* is the protein families where the node *n* is present, and *siblings(n)* are the set of nodes which are the siblings of *n* in the phylogenetic tree.

Loss of a family in the course of evolution from the most recent common ancestor is defined as the set of protein families that exist both in one of node’s uncles and in one of its siblings but do not exist for the node. The set of lost families for the node *n* is defined by the following formula (with the same definitions as for gain):

$$ loss(n)=\left[{\displaystyle \underset{s\in siblings(n)}{\cup }pf(s){\displaystyle \cap {\displaystyle \underset{u\in siblings\left( father(n)\right)}{\cup }pf(u)}}}\right]\backslash pf(n) $$

### Turnover rate estimation

The turnover rate (TOR) of a node in the phylogenetic tree is defined as the sum of gains and losses divided by the length of the branch to its ancestor. The TOR of species is defined as the sum of TOR values of its ancestors. Losses could be defined more conservatively, by considering only families that have maintained all insect species representatives except the subjected one (described in [62]). A threshold of 50 proteins in a family was defined to secure a strict definition for family loss. Estimation of TOR using BadiRate [26] could not be directly applied on our data due to the requirement for binary phylogenetic tree, while the considered tree has one node with three direct children and one node with four (see Fig. 2). Therefore, we imposed on the phylogenetic tree a binary structure by eliminating branches. We eliminated one or two branches (from nodes with out-degree of 3 and 4, respectively) to produce all combinations of 12 different binary trees. BadiRate parameters used are: -bmodel FR -rmodel BDI -ep CSP.

### Expansion and reduction of protein families

Statistical significance of expansion or contraction of a protein family of species was computed according to hypergeometric *P*-value. Expansion’s *P*-value was computed using the cumulative distribution:

$$ p-value\left(x,n\right)=\sum_{k=x}^n\frac{\left(\kern1em \begin{array}{c}K\kern1em \\ {}\kern1em i\end{array}\right)\kern1em \left(\kern1em \begin{array}{c}N-K\kern1em \\ {}\kern1em n-i\end{array}\kern1em \right)}{\left(\kern1em \begin{array}{c}N\kern1em \\ {}\kern1em n\end{array}\kern1em \right)} $$

Where *K* is the number of proteins of that species, *x* is the number of proteins of that species in the given protein family, *N* is the total number of proteins, and *n* is the protein family size (i.e., total number of proteins). The value of this sum is simply the hypergeometric probability mass function, and we sum from *x* up to the size of the protein family to estimate the probability that one species will have *x* or more proteins in this protein family. We applied the Benjamini & Hochberg FDR correction [63] to account for multiple hypothesis testing. The *P*-value for contraction is very similar, but the sum ranges from 0 to *x*. This is the probability of having no more than *x* proteins in the specific protein family for a given species.

## Availability of supporting data

Additional file 1: Tables S1, Additional file 2: Tables S2, Additional file 3: Tables S3, Additional file 4: Tables S4 and Additional file 5: Tables S5 are available in http://protobug.cs.huji.ac.il/BMC-G_supp_tables/.

## Additional files

Additional file 1: Table S1. Data for 17 proteomes from insects and an additional proteome from the crustacean *D. pulex.*
Additional file 2: Table S2. Comprehensive list of 3437 mapped Pfam keywords (associated with 4503 ProtoBug families). Additional file 3: Table S3. A comprehensive list of the families marked as Gain or Loss. Additional file 4: Table S4. A list of the most significant expanded and contracted species-specific families (SSF) per 18 species. Additional file 5: Table S5. A list of the 114 Root SFs that contains >200 proteins from Hymenoptera or Diptera. The dominating annotations and additional proteins in the SF are listed.

## Abbreviations

ECM: Extracellular Matrix
FDR: False Discovery Rate
GO: Gene Ontology
MR: Membrane Receptors
SF: Superfamily
SSF: Species-Specific Families
TNAP: Transposition and Nucleic Acids Process
TOR: Turnover rate
TM: Transmemberane
