# Supplementary material for: Trends in genome dynamics among major orders of insects revealed through variations in protein families
Source: BMC Genomics. 2015 Aug 7;16(1):583. doi: 10.1186/s12864-015-1771-2 (PMC4528696; doi:10.1186/s12864-015-1771-2)
Supplement: Additional file 1: Table S1. — Data for 17 proteomes from insects and an additional proteome from the crustacean D. pulex. [file 12864_2015_1771_MOESM1_ESM.docx]

Additional File 1: Table S1.

Sources for all 18 complete proteomes and the number of protein families from the PL70 partition. There are 17 proteomes from insects and one proteome from Crustacean (*D. pulex*).

| **Taxa-ID** | **Organism (common name)** | **DB Source**^a^ | **#Proteins** | **#Families** |
| --- | --- | --- | --- | --- |
| [7070](http://www.uniprot.org/taxonomy/7070) | Tribolium castaneum (Red flour beetle) | UniProt | [16502](http://www.uniprot.org/uniprot/?query=taxonomy:7070+AND+keyword:181) | 6706 |
| [7159](http://www.uniprot.org/taxonomy/7159) | Aedes aegypti (Yellowfever mosquito) | UniProt | [16045](http://www.uniprot.org/uniprot/?query=taxonomy:7159+AND+keyword:181) | 5120 |
| [7165](http://www.uniprot.org/taxonomy/7165) | Anopheles gambiae (African malaria mosquito) | UniProt | [13075](http://www.uniprot.org/uniprot/?query=taxonomy:7165+AND+keyword:181) | 4563 |
| [7176](http://www.uniprot.org/taxonomy/7176) | Culex quinquefasciatus (Southern house mosquito) | UniProt | [18703](http://www.uniprot.org/uniprot/?query=taxonomy:7176+AND+keyword:181) | 5415 |
| [7227](http://www.uniprot.org/taxonomy/7227) | Drosophila melanogaster (Fruit fly) | UniProt | [17524](http://www.uniprot.org/uniprot/?query=taxonomy:7227+AND+keyword:181) | 5233 |
| [7244](http://www.uniprot.org/taxonomy/7244) | Drosophila virilis (Fruit fly) | UniProt | [14457](http://www.uniprot.org/uniprot/?query=taxonomy:7244+AND+keyword:181) | 5662 |
| [7425](http://www.uniprot.org/taxonomy/7425) | Nasonia vitripennis (Parasitic wasp) | [HGD (v1.2)](http://www.hymenopteragenome.org/nasonia/?q=sequencing_and_analysis_consortium_datasets) | 18822 | 5058 |
| [13686](http://www.uniprot.org/taxonomy/13686) | Solenopsis invicta (Red imported fire ant) | UniProt | [14194](http://www.uniprot.org/uniprot/?query=taxonomy:13686+AND+keyword:181) | 5814 |
| [43151](http://www.uniprot.org/taxonomy/43151) | Anopheles darlingi (Mosquito) | UniProt | [11437](http://www.uniprot.org/uniprot/?query=taxonomy:43151+AND+keyword:181) | 5446 |
| [103372](http://www.uniprot.org/taxonomy/103372) | Acromyrmex echinatior (Panamanian leafcutter ant) | UniProt | [13962](http://www.uniprot.org/uniprot/?query=taxonomy:103372+AND+keyword:181) | 6538 |
| [104421](http://www.uniprot.org/taxonomy/104421) | Camponotus floridanus (Florida carpenter ant) | UniProt | [14787](http://www.uniprot.org/uniprot/?query=taxonomy:104421+AND+keyword:181) | 6008 |
| [121224](http://www.uniprot.org/taxonomy/121224) | Pediculus humanus subsp. corporis (Body louse) | UniProt | [10763](http://www.uniprot.org/uniprot/?query=taxonomy:121224) | 4866 |
| [610380](http://www.uniprot.org/taxonomy/610380) | Harpegnathos saltator (Jumping Ant) | UniProt | [15029](http://www.uniprot.org/uniprot/?query=taxonomy:610380) | 5423 |
| [7460](http://www.uniprot.org/taxonomy/7460) | Apis mellifera (Honeybee) | BeeBase (v4.5) | 10570 | 4521 |
| [83485](http://www.uniprot.org/taxonomy/83485) | Linepithema humile (Argentine ant) | [HGD (v1.2)](http://www.hymenopteragenome.org/nasonia/?q=sequencing_and_analysis_consortium_datasets) | 16116 | 6964 |
| [12957](http://www.uniprot.org/taxonomy/12957) | Atta cephalotes (Leaf Cutter Ant) | [HGD (v1.2)](http://www.hymenopteragenome.org/nasonia/?q=sequencing_and_analysis_consortium_datasets) | 18093 | 8378 |
| [144034](http://www.uniprot.org/taxonomy/144034) | Pogonomyrmex barbatus (Red Harvester Ant) | [HGD (v1.2)](http://www.hymenopteragenome.org/nasonia/?q=sequencing_and_analysis_consortium_datasets) | 17189 | 7999 |
| [6669](http://www.uniprot.org/taxonomy/6669) | Daphnia pulex (water flea) | UniProt | [30137](http://www.uniprot.org/uniprot/?query=taxonomy:6669+AND+keyword:181) | 8742 |

^a^Source of BeeBase belong to HGD, Hymenoptera Genomic Database.
